# Predictive modeling of lung cancer subtype with integration of clinical and thoracic imaging features to guide early brain metastasis management

**DOI:** 10.1007/s11060-026-05812-z

**Published:** 2026-10-05

**Authors:** Jino Park, Paul Buclez, Jessica Lubisich, Krishna Hanubal, Andrea Inda, Caressa Hui, Jeremy Harris, Aaron Simon

**Affiliations:** 1 Department of Radiation Oncology, Radiation Oncology Resident, UC Irvine Department of Radiation Oncology, University of California Irvine, 101 The City Dr. S, Building 23, Orange, CA USA; 2 California Northstate University College of Medicine, Elk Grove, CA USA; 3 University of California Irvine School of Medicine, Irvine, CA USA

**Keywords:** Brain metastasis, EGFR, Non-small cell lung cancer, Small cell lung cancer, Predictive modeling, Machine learning

## Abstract

**Purpose:**

Optimal treatment for synchronous brain metastases at lung cancer diagnosis depends on subtype, but histologic/molecular profiling is often delayed. We developed machine-learning models using clinical and imaging data to classify lung cancer into three subtypes with distinct brain metastasis treatment implications: small cell lung cancer (SCLC), EGFR-mutated non-small cell lung cancer (EGFR+), and EGFR–wild-type non-small cell lung cancer (EGFR-).

**Methods:**

We retrospectively identified patients with metastatic lung cancer diagnosed from 2016 to 2025 at a single institution. Patients without brain metastases at diagnosis formed the training cohort, while patients presenting with synchronous brain metastases formed the testing cohort. Clinical variables and thoracic imaging features from diagnostic chest CT reports were used to train LASSO logistic regression and random forest models, evaluated via AUC and standard diagnostic metrics. Feature contributions were assessed using logistic regression coefficients and random forest permutation importance.

**Results:**

Of 305 patients, 182 were used for training and 123 comprised the testing cohort. Logistic regression achieved AUCs of 0.887, 0.811, and 0.801 for EGFR+, SCLC, and EGFR-, respectively; random forest achieved 0.907, 0.783, and 0.810. Adding imaging features to clinical variables improved prediction for EGFR + and SCLC. Contributory imaging features included fibrosis, emphysema, miliary pattern, cavitation, pleural attachment, and vessel encasement, among others. Three-way prediction showed highest positive predictive value for EGFR + and high negative predictive value for SCLC.

**Conclusion:**

Combined clinical and chest imaging features can help predict lung cancer subtype, supporting early multidisciplinary discussions for patients with synchronous brain metastases.

**Supplementary Information:**

The online version contains supplementary material available at https://doi.org/10.1007/s11060-026-05812-z.

## Introduction

Brain metastases represent a pressing oncologic development, requiring urgent management to prevent highly morbid and potentially irreversible sequelae. Decision making can be particularly challenging when patients present with synchronous brain metastases at the time of a new lung cancer diagnosis, as the optimal treatment strategy may depend on tumor histology and molecular subtype. For example, except in true emergencies, patients with small cell lung cancer (SCLC) may derive limited benefit from surgical resection owing to SCLC’s exquisite radiosensitivity [1–4]. Conversely, patients with large tumors from non-small cell lung cancer (NSCLC) may benefit from surgical resection prior to radiotherapy to reduce mass effect and improve local control [5–7]. Meanwhile, select patients with subsets of NSCLC harboring driver mutations may be able to defer upfront radiotherapy to trial a tyrosine kinase inhibitor (TKI) with intracranial activity [8–13].

Prompt identification of lung cancer subtype is critical to optimizing brain metastasis management. However, time required to obtain biopsies, process pathological specimens, and assess for actionable biomarkers can delay diagnosis by days to weeks [14, 15]. In this context, a predictive tool capable of estimating lung cancer subtype using readily available clinical and radiographic features could provide meaningful guidance for early treatment planning and multidisciplinary discussion. In the present study, we developed predictive models integrating clinical and thoracic computed tomography (CT) imaging features to classify lung cancer subtype among three clinically relevant groups: SCLC, EGFR-mutated NSCLC (EGFR+), and EGFR–wild-type NSCLC (EGFR-).

## Methods

### Study cohort

The study was approved by the institutional review board. The records of patients diagnosed with metastatic lung cancer between 2016 and 2025 were retrospectively obtained from a single institutional database. Those with initially localized disease or who received prior cancer treatment were excluded. Eligible patients were required to have available radiology reports of the initial diagnostic chest CT with or without contrast and brain MRI with and without contrast performed within two months of diagnosis, as well as documented histologic and molecular classification of lung cancer subtype. Patients were categorized into three groups according to final histologic and molecular diagnoses: SCLC, EGFR+, and EGFR-. Of all possible driver mutations, only EGFR was selected for independent categorization because (1) with exception of KRAS, it is significantly more prevalent than other driver mutations and (2) unlike KRAS, multiple CNS penetrating targeted therapies are approved for EGFR+ patients [16–19].

### Data partitioning

Patients without brain metastases at diagnosis were used for model development, while patients presenting with synchronous brain metastases were reserved as a held-out internal testing cohort. This partitioning strategy was selected to maximize the number of patients available for model training while preserving the entire clinically relevant target population of patients, those with synchronous brain metastases, for testing. The intended application of the model is therefore early probabilistic subtype estimation specifically in patients presenting with synchronous brain metastases while definitive histologic and molecular characterization is pending. Because patients with and without brain metastases at presentation may differ in clinical and imaging characteristics, this partitioning strategy introduces the potential for distribution shift between the training and testing populations, which was evaluated in supplemental sensitivity analyses.

### Clinical and imaging feature selection

Variables that may help distinguish lung cancer subtypes were extracted from the electronic medical record. These included baseline clinical information and radiographic features reported in literature to be associated with SCLC or EGFR+. ^1,20–28^ Clinical variables included sex, age at diagnosis, Asian race, white race, smoking history, pack-years, and prior history of lung disease such as emphysema, chronic obstructive pulmonary disease or interstitial lung disease. Thoracic imaging features were obtained from the initial diagnostic chest CT reports. To standardize extraction from narrative reports, an institutional, firewalled large language model (LLM) (Anthropic Claude Sonnet 4.6) blinded to patient identifiers and tumor subtype was used to determine the presence or absence of the following predefined imaging features: location (central vs. peripheral), pleural attachment, cavitation, lymphangitic spread, endobronchial component, fibrosis, miliary pattern, emphysema, T3 or T4 classification, multifocality, mediastinal lymphadenopathy, bulky nodal involvement, vessel encasement, superior vena cava (SVC) involvement, and osseous metastases. Findings explicitly described as present, as well as equivocal statements indicating possible presence (e.g. “cannot exclude”), were coded as present. Findings explicitly described as absent or not mentioned in the report were coded as absent. The standardized prompt engineered to extract imaging features is shown in supplemental document 1.

To validate the LLM-based imaging feature extraction process, 50 randomly selected chest CT reports were manually reviewed by the investigators using the same aforementioned imaging features. Human annotation was used as the reference standard, performed blinded to the LLM output, and assessed by percent agreement and Cohen’s kappa for each imaging feature.

### Model development and testing

Two machine learning approaches were evaluated to predict tumor subtype. The first approach used logistic regression with least absolute shrinkage and selection operator (LASSO) regularization, which allows for feature selection and reduces overfitting by penalizing less informative variables. The second approach employed a random forest classifier, an ensemble learning method that constructs multiple decision trees and aggregates predictions to improve classification performance. To determine the incremental predictive value of incorporating imaging features, the models were developed and tested using two different feature sets. The first included clinical variables alone, and the second incorporated both clinical and imaging features.

A one-versus-rest classification strategy was used to evaluate model performance for each lung cancer subtype. Model performance was assessed using the area under the receiver operating characteristic curve (AUC) as the primary metric of discrimination. 95% confidence intervals for testing AUCs were estimated using 2,000 bootstrap resamples. Predictors with nonzero LASSO coefficients at the selected penalty were entered into a multivariable logistic regression model using Firth penalized likelihood. This secondary analysis was performed to provide interpretable effect estimates for the retained predictors and are reported as exploratory measures of association rather than confirmatory inference. Random forest feature importance was assessed using out-of-bag permuted predictor delta error, defined as the increase in out-of-bag prediction error after randomly permuting each predictor, with larger values indicating greater contribution to model performance. Paired DeLong tests were used to compare AUCs between clinical only and combined clinical and imaging models and between LASSO logistic regression and random forest models evaluated in the same testing cohort.

Positive predictive value (PPV), negative predictive value (NPV), sensitivity, specificity, F1 score, overall accuracy, and Cohen’s kappa were calculated to characterize classification performance. Because the subtype specific AUC analyses were performed using one-versus-rest models, separate multiclass models were developed to evaluate three-way classification. For logistic regression, a nominal multinomial logistic regression model was trained to classify EGFR+, SCLC, and EGFR-. For random forest, a single multiclass random forest was trained using the same three mutually exclusive outcome categories. Both multiclass models generated a joint probability distribution across the three subtypes for each patient, with probabilities summing to 1. Patients were then assigned to the subtype with the highest predicted probability.

To evaluate clinical utility beyond discrimination and classification accuracy, an exploratory decision curve analysis was performed in the held-out testing cohort using the predicted class probabilities generated by the multinomial logistic regression and multiclass random forest models. For each subtype, net benefit was calculated across threshold probabilities ranging from 0.05 to 0.80. The threshold probability represents the minimum predicted probability at which a clinician would act on a predicted subtype, for example by flagging a patient for expedited histologic or molecular confirmation. Model net benefit was compared against two reference strategies, flagging all patients and flagging no patients.

**Fig. 1:**
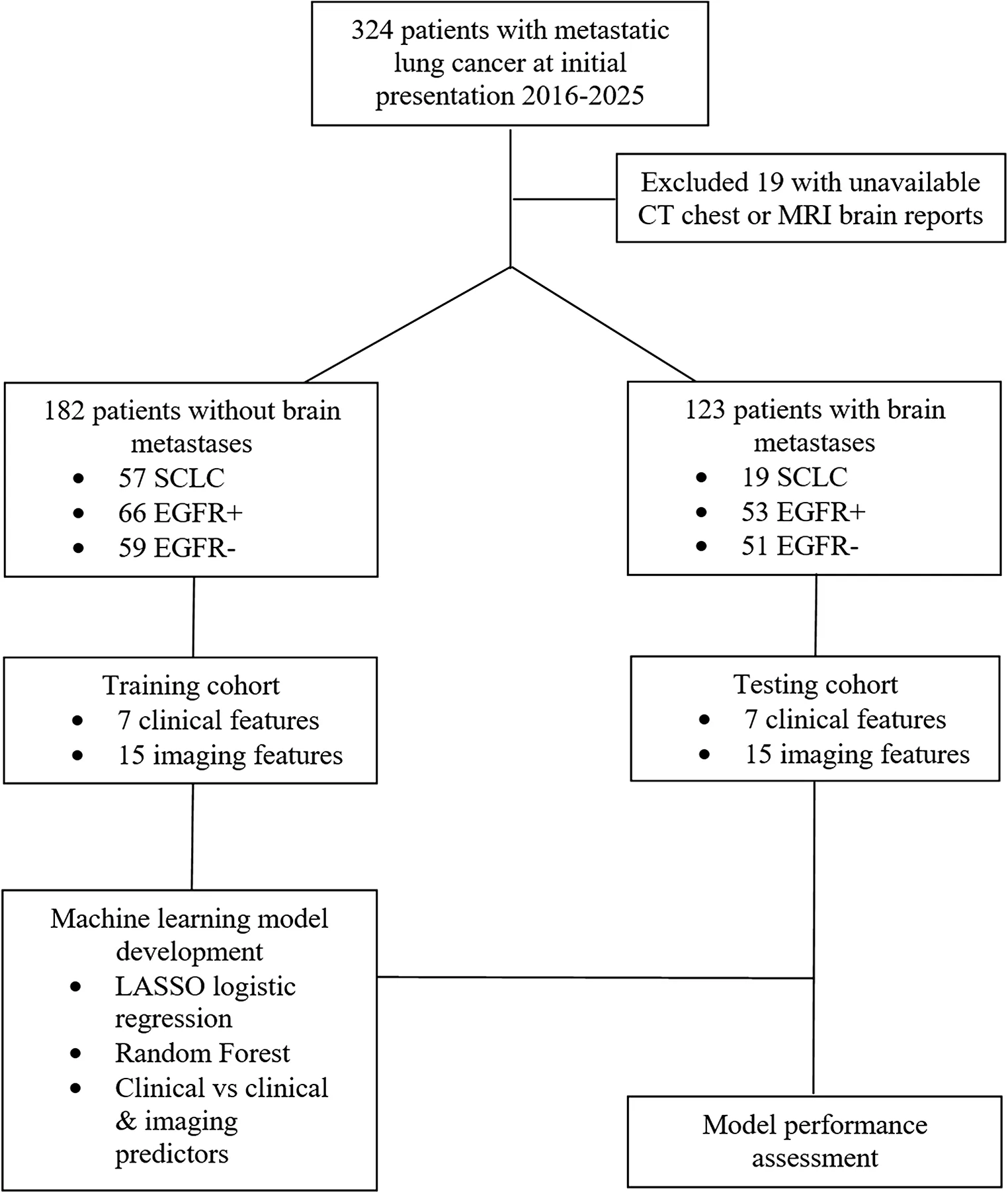
Patient selection and study design

Machine learning models were developed and evaluated using MATLAB R2025b (The MathWorks Inc., Natick, MA, USA), the Statistics and Machine Learning Toolbox, and the Classification Learner framework for model training, cross-validation, and performance evaluation. Additional statistical analyses and were performed using R version 4.5.2 (R Foundation for Statistical Computing, Vienna, Austria). Machine learning specifications are shown in Supplemental Table 7. A simplified, overview study design is shown in Fig. 1.

### Data partitioning validation and covariate shift sensitivity analyses

Following the completion of the primary analysis, additional sensitivity analyses were performed to evaluate the potential impact of the cohort partitioning strategy. First, we performed a supplemental size-matched sensitivity analysis to assess whether training on patients without brain metastases substantially altered model discrimination when applied to patients with synchronous brain metastases. Sixty patients with brain metastases were randomly selected and held out as a common testing cohort. Models were then trained using either 60 additional patients with brain metastases or 60 randomly selected patients without brain metastases. Testing AUCs for EGFR+, SCLC, and EGFR- classification were compared between the two training strategies using paired DeLong tests.

We additionally evaluated potential covariate shift between the full training and testing cohorts. Clinical and imaging characteristics that differed significantly between the overall cohorts were first compared within each lung cancer subtype using Fisher’s exact tests to rule out variations within each subtype. To further assess differences in the overall covariate distribution, we performed a reweighting sensitivity analysis using all baseline clinical and imaging predictors. The probability of belonging to the testing cohort was estimated from baseline covariates, and training observations were assigned inverse-odds weights to better approximate the covariate distribution of the testing population. Covariate balance before and after weighting was assessed using absolute standardized mean differences (SMD), with an absolute SMD < 0.10 indicating adequate balance. LASSO logistic regression and random forest models were then refit using the weighted training cohort and evaluated on the unchanged held-out testing cohort. Weighted and unweighted models were compared using the same model-fitting approach to assess changes in testing AUC after covariate reweighting.

## Results

### Study cohort and baseline characteristics

A total of 324 patients with metastatic lung cancer at initial presentation were identified from 2016 to 2025. After exclusion of 19 patients without available chest CT or brain MRI reports, 305 patients were included in the final analysis. Patients without brain metastases at diagnosis were used for model training and included 182 patients, consisting of 57 patients with SCLC, 66 with EGFR+, and 59 with EGFR-. Patients with brain metastases at diagnosis were used as the testing cohort and included 123 patients, consisting of 19 patients with SCLC, 53 with EGFR+, and 51 with EGFR-.

Baseline characteristics of the training and testing cohorts are shown in Table 1. The two cohorts were generally well balanced across most clinical variables. The training and testing cohorts had similar sex distribution, with women comprising 46% and 44% of patients, respectively. The mean age was 65.6 years in the training cohort and 63.1 years in the testing cohort. Smoking history was present in 64% of the training cohort and 58% of the testing cohort, and mean pack-year history was 22.5 and 18.6, respectively. Prior lung disease was more common in the training cohort than the testing cohort, occurring in 51% versus 39% of patients. Most imaging features were also similar between cohorts. Cavitation was more common in the testing cohort than the training cohort, occurring in 17% versus 7% of patients, while fibrosis was less common in the testing cohort, occurring in 15% versus 25%.

**Table 1:** Baseline clinical and imaging characteristics of the training and testing cohorts

Variable	Training (*n* = 182)	Testing (*n* = 123)	*p*-value
**Lung Cancer Subtype**			
SCLC	57	19	
EGFR+	66	53	
EGFR-	59	51	
**Clinical**			
**Sex**			0.769
Female (%)	83 (46)	54 (44)	
Male (%)	99 (54)	69 (56)	
**Age**,** y**			0.089
Mean (SD)	65.6 (9.3)	63.1 (11.1)	
Median (Range)	66 (42–99)	64 (18–90)	
**Race**			0.055
White (%)	82 (45)	40 (33)	
Asian (%)	72 (40)	65 (53)	
Other (%)	28 (15)	18 (15)	
**Smoking history**			0.248
Yes (%)	117 (64)	71 (58)	
No (%)	65 (36)	52 (42)	
**Pack-years**			0.201
Mean (SD)	22.5 (25)	18.6 (22)	
**Prior lung disease**			0.038
Yes (%)	93 (51)	48 (39)	
No (%)	89 (49)	75 (61)	
**Imaging**			
**Location**			0.456
Central (%)	78 (43)	44 (36)	
Peripheral (%)	42 (23)	33 (27)	
Unspecified (%)	62 (34)	46 (37)	
**Pleural attachment**			0.744
Yes (%)	75 (41)	53 (43)	
No (%)	107 (59)	70 (57)	
**Cavitation**			0.004
Yes (%)	12 (7)	21 (17)	
No (%)	170 (93)	102 (83)	
**Lymphangitic spread**			0.439
Yes (%)	47 (26)	27 (22)	
No (%)	135 (74)	96 (78)	
**Endobronchial component**			0.115
Yes (%)	31 (17)	30 (24)	
No (%)	151 (83)	93 (76)	
**Fibrosis**			0.033
Yes (%)	45 (25)	18 (15)	
No (%)	137 (75)	105 (85)	
**Miliary**			0.166
Yes (%)	9 (5)	11 (9)	
No (%)	173 (95)	112 (91)	
**Emphysema**			0.182
Yes (%)	54 (30)	28 (23)	
No (%)	128 (70)	95 (77)	
**T3 or T4**			0.468
Yes (%)	128 (70)	81 (66)	
No (%)	54 (30)	42 (34)	
**Multifocal**			0.304
Yes (%)	120 (66)	74 (60)	
No (%)	62 (34)	49 (40)	
**Mediastinal lymphadenopathy**			0.809
Yes (%)	112 (61)	74 (60)	
No (%)	70 (39)	49 (40)	
**Bulky nodal involvement**			0.432
Yes (%)	63 (35)	48 (39)	
No (%)	119 (65)	75 (61)	
**Vessel encasement**			0.743
Yes (%)	40 (22)	29 (24)	
No (%)	142 (78)	94 (76)	
**superior vena cava involvement**			0.533
Yes (%)	12 (7)	6 (5)	
No (%)	170 (93)	117 (95)	
**Osseous Metastases**			0.321
Yes (%)	44 (24)	36 (29)	
No (%)	138 (76)	87 (71)	

### Validation of covariate shift and LLM utilization

Because prior lung disease, fibrosis, and cavitation differed significantly between the overall training and testing populations, we evaluated these variables after stratification by lung cancer subtype to assess whether the observed differences were primarily attributable to differences in subtype composition. No statistically significant differences were detected for any of these three characteristics within the EGFR+, SCLC, or EGFR- strata by Fisher’s exact test (Supplemental Table 1). Potential covariate distribution shift risk was further evaluated using a reweighting analysis. Before weighting, 9 of 22 predictors demonstrated an absolute SMD < 0.10, with a mean absolute SMD of 0.141 and maximum absolute SMD of 0.328. After reweighting the training cohort toward the covariate distribution of the testing population, 21 of 22 predictors demonstrated an absolute SMD < 0.10, and the mean and maximum absolute SMDs decreased to 0.042 and 0.108, respectively (Supplemental Fig. 1). Despite this improvement in covariate balance, model discrimination remained similar after reweighting. Across the six model and subtype comparisons, the mean absolute change in testing AUC was 0.010 and the maximum absolute change was 0.024 for the logistic regression SCLC model (Supplemental Table 2). In the size-matched sensitivity analysis, no statistically significant differences in testing AUC were detected between models trained using patients with versus without brain metastases (Supplemental Table 3). However, point estimates differed for some comparisons, particularly for SCLC, and the relatively small sample size and class imbalance limited statistical power.

The fidelity of LLM imaging feature extraction was evaluated against human manual reference in 50 diagnostic chest CT reports, representing 750 total individual feature comparisons. Overall agreement between the LLM output and human reference features was 96.8%. Agreement was lowest for identifying T3 or T4 classification at 90%. Cohen’s kappa ranged from 0.694 to 1.000 across the 15 imaging features with an overall value of 0.918 (Supplemental Table 4).

### Model performance

**Fig. 2:**
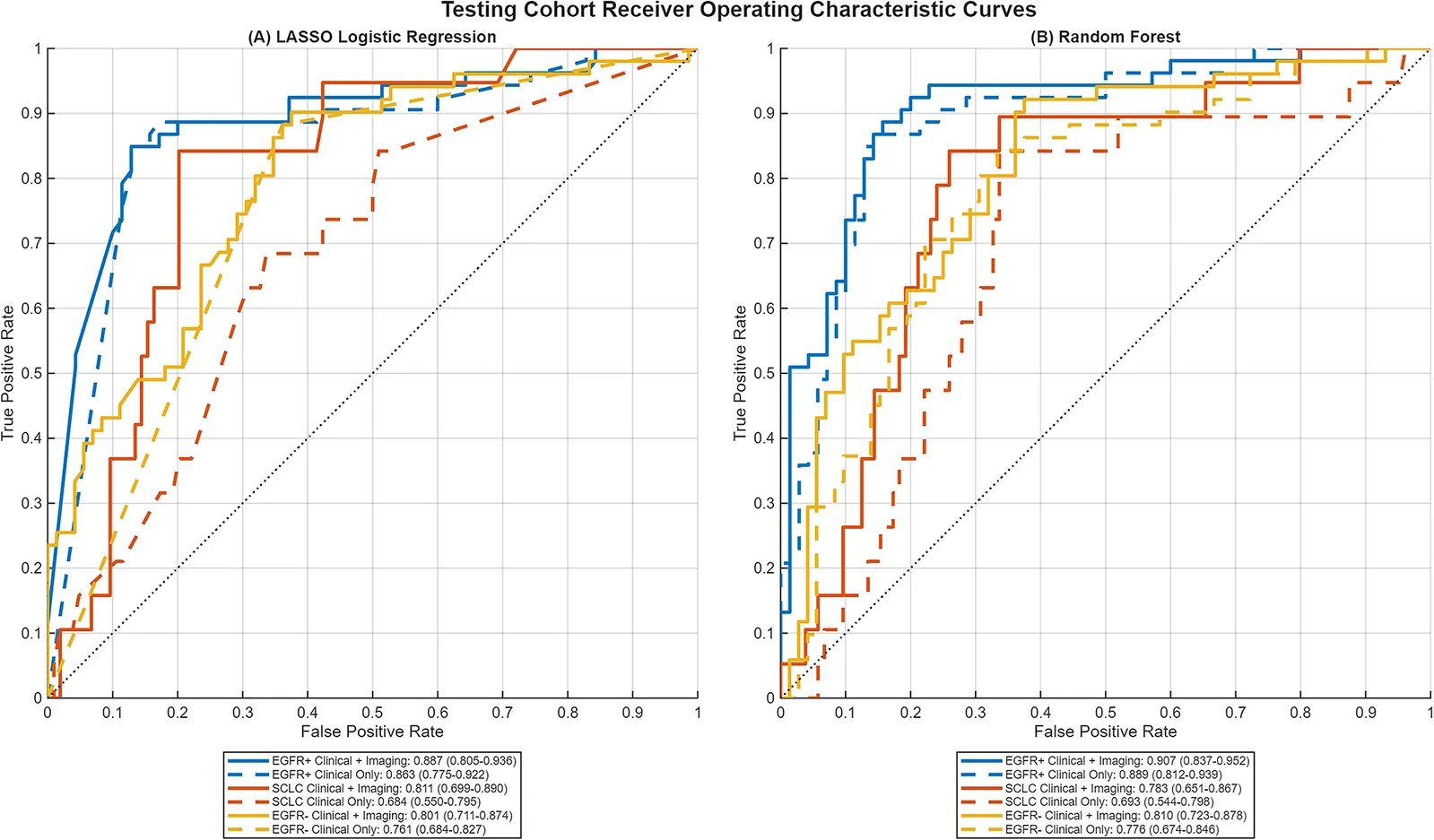
Testing cohort receiver operating characteristic curves for **(A)** LASSO logistic regression and **(B)** random forest models. Dashed curves represent models using clinical variables alone, while solid curves represent models incorporating both clinical and imaging features. Testing AUCs and corresponding 95% bootstrap confidence intervals are shown in legends

In the primary analysis, LASSO logistic regression and random forest models were trained using the cohort without brain metastases and tested in the held-out internal cohort with synchronous brain metastases. Models were evaluated using both clinical variables alone and combined clinical plus imaging variables. Across both modeling approaches, incorporation of imaging features generally improved discrimination compared with clinical variables alone, as shown in Fig. 2. For the LASSO logistic regression model, the combined clinical and imaging model achieved testing AUCs of 0.887 (95% CI, 0.805–0.936) for EGFR+, 0.811 (95% CI, 0.699–0.890) for SCLC, and 0.801 (95% CI, 0.711–0.874) for EGFR-. In comparison, the clinical-only LASSO model achieved testing AUCs of 0.863 (95% CI, 0.775–0.922), 0.684 (95% CI, 0.550–0.795), and 0.761 (95% CI, 0.684–0.827), respectively. Addition of imaging features significantly improved discrimination for EGFR+ (DeLong *p* = 0.040) and SCLC (*p* = 0.009), but not for EGFR- (*p* = 0.134). For random forest, the combined clinical and imaging model achieved testing AUCs of 0.907 (95% CI, 0.837–0.952) for EGFR+, 0.783 (95% CI, 0.651–0.867) for SCLC, and 0.810 (95% CI, 0.723–0.878) for EGFR-. The corresponding clinical only AUCs were 0.889 (95% CI, 0.812–0.939), 0.693 (95% CI, 0.544–0.798), and 0.776 (95% CI, 0.674–0.846). Addition of imaging features significantly improved discrimination for SCLC (DeLong *p* = 0.016), while differences for EGFR+ (*p* = 0.087) and EGFR− (*p* = 0.137) did not reach statistical significance. Overall, the random forest and LASSO models demonstrated similar performance (DeLongs test, EGFR + *p* = 0.291; SCLC *p* = 0.271; EGFR- *p* = 0.572), while the highest discrimination was observed for the EGFR+ subtype.

**Fig. 3:**
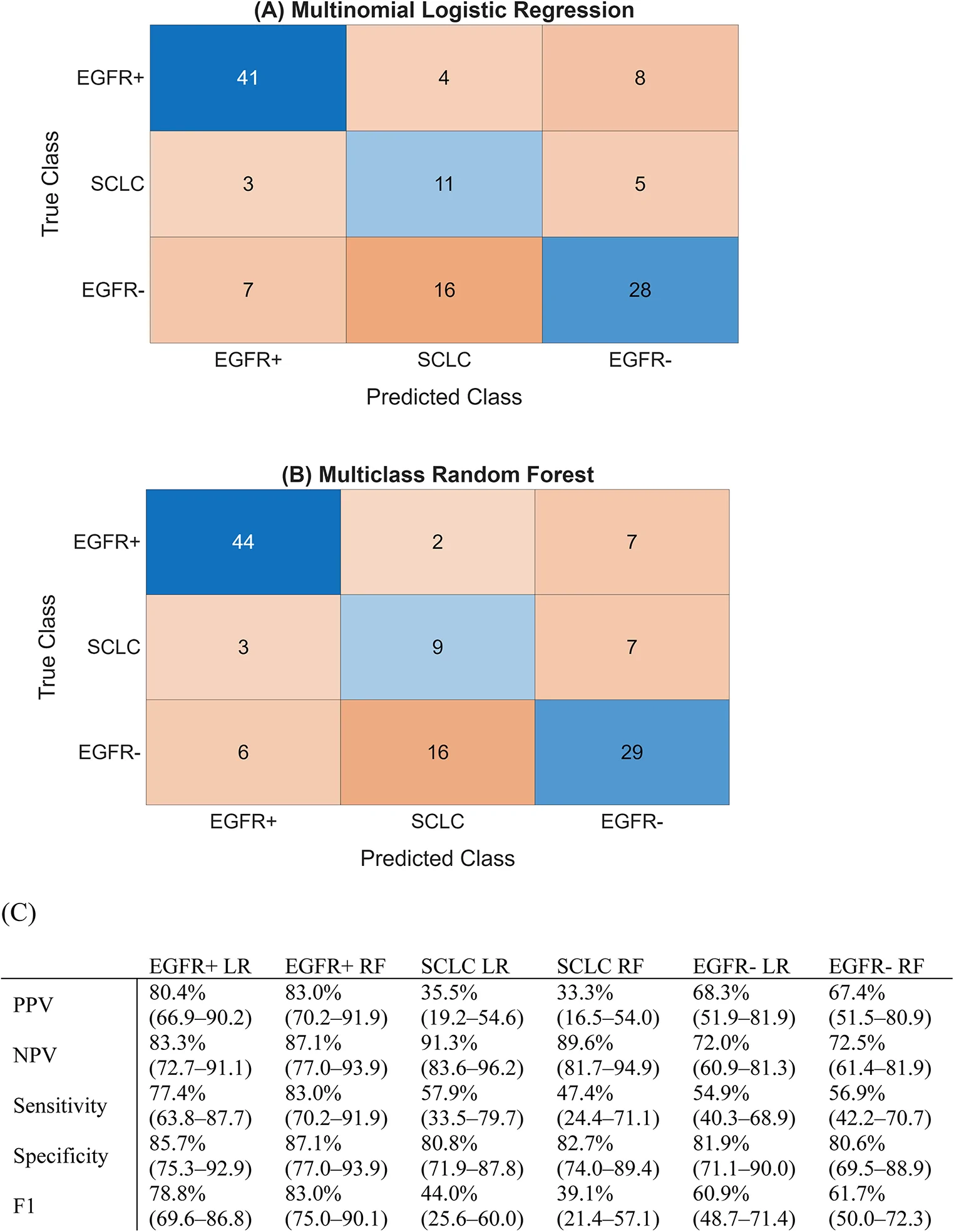
Three-way lung cancer subtype classification in the testing cohort using (A) multinomial logistic regression and (B) multiclass random forest. Each patient assigned to the subtype with the highest predicted class probability. (C) shows corresponding performance metrics

To assess three-way classification performance, separate multinomial logistic regression model and a multiclass random forest model were trained to classify patients as EGFR+, SCLC, or EGFR-. The multinomial logistic regression model achieved PPVs of 80.4%, 35.5%, and 68.3% for EGFR+, SCLC, and EGFR-, respectively, with corresponding NPVs of 83.3%, 91.3%, and 72.0%. The overall accuracy was 65.0%, a macro F1 score of 0.612, and a Cohen’s kappa of 0.457. The multiclass random forest achieved a similar overall accuracy of 66.7%, with a macro F1 score of 0.613 and Cohen’s kappa of 0.475. PPVs were 83.0%, 33.3%, and 67.4% for EGFR+, SCLC, and EGFR-, respectively, with corresponding NPVs of 87.1%, 89.6%, and 72.5% (Fig. 3 and Supplemental Table 5).

**Fig. 4:**
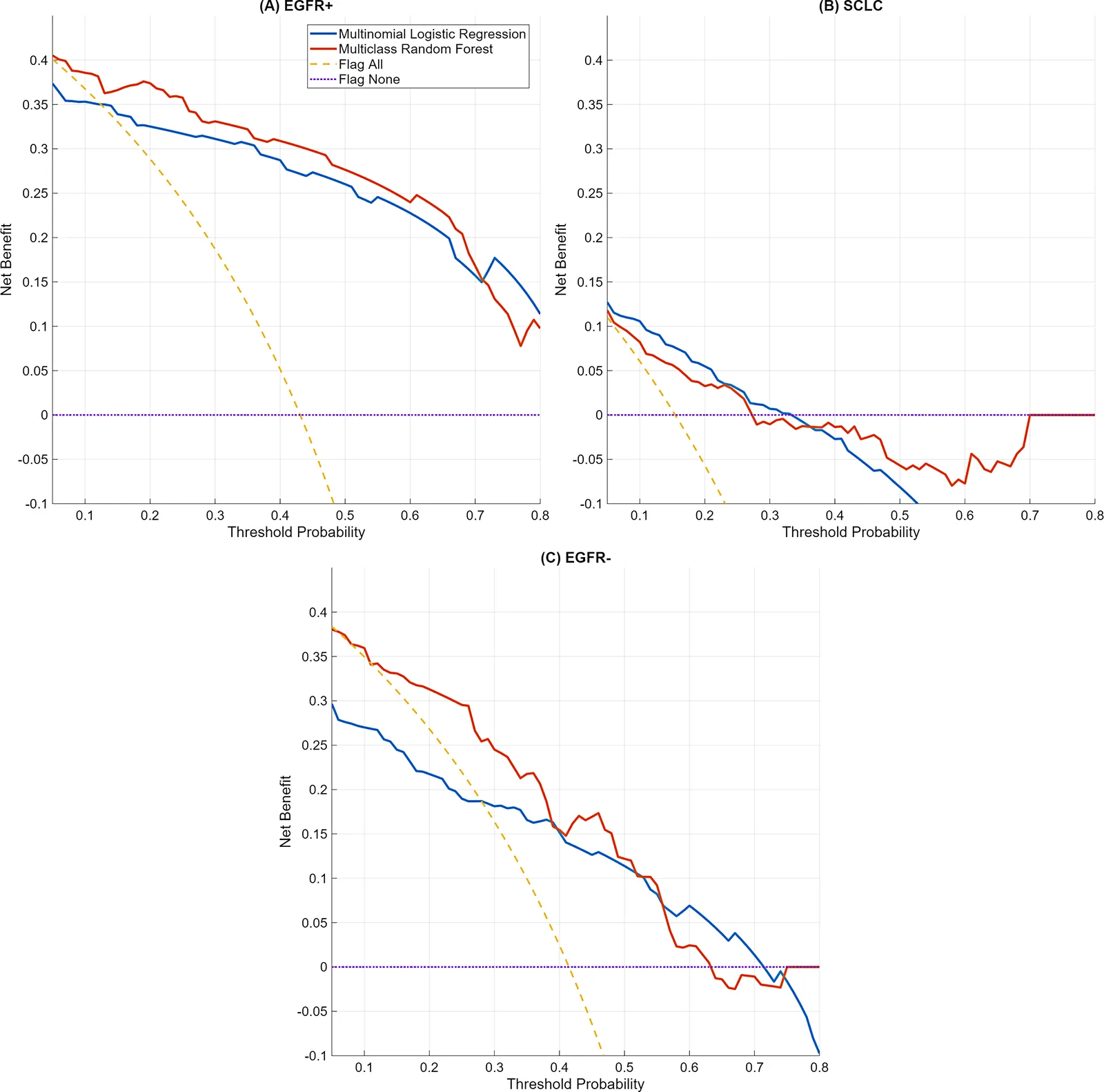
Decision curve analysis in the testing cohort for **(A)** EGFR+, **(B)** SCLC, and **(C)** EGFR-. Net benefit is plotted against threshold probability for the multinomial logistic regression model and the multiclass random forest model. Threshold probability represents the minimum predicted probability at which a clinician would act on the predicted subtype, for example by flagging a patient for expedited pathologic or molecular confirmation. Clinical utility differed by subtype: both models provided net benefit for EGFR+ across essentially the full range of thresholds examined, whereas for SCLC benefit was confined to low thresholds

Decision curve analysis was performed in the held-out testing cohort to evaluate the clinical utility of the multiclass models across a range of decision thresholds (Fig. 4). For EGFR+, the multiclass random forest demonstrated greater net benefit than both the flag-all and flag-none reference strategies across the entire threshold range examined, from 0.05 to 0.80, while the multinomial logistic regression model exceeded both reference strategies from a threshold probability of 0.13 to 0.80. At a representative threshold probability of 0.30, net benefit was 0.311 for multinomial logistic regression and 0.331 for multiclass random forest, compared with 0.187 for the flag-all strategy, corresponding to approximately 12 and 14 additional correct EGFR+ identifications per 100 patients evaluated, respectively, without a corresponding increase in false positive classifications. For EGFR-, the multiclass random forest exceeded both reference strategies from a threshold probability of 0.06 to 0.63, and the multinomial logistic regression model from 0.28 to 0.71. At a threshold probability of 0.40, added net benefit was 0.127 for multinomial logistic regression and 0.130 for multiclass random forest. For SCLC, net benefit exceeded both reference strategies only at lower threshold probabilities, from 0.05 to 0.33 for multinomial logistic regression and from 0.05 to 0.27 for multiclass random forest. At a threshold probability of 0.20, added net benefit for SCLC was 0.055 for multinomial logistic regression and 0.033 for multiclass random forest, whereas net benefit was negative for both models at threshold probabilities of 0.40 and above.

An exploratory analysis of the six patients who were EGFR- but incorrectly classified as EGFR + in the RF model showed that four harbored other driver mutations associated with non-smoking related lung cancer (Supplemental Table 6).

**Fig. 5:**
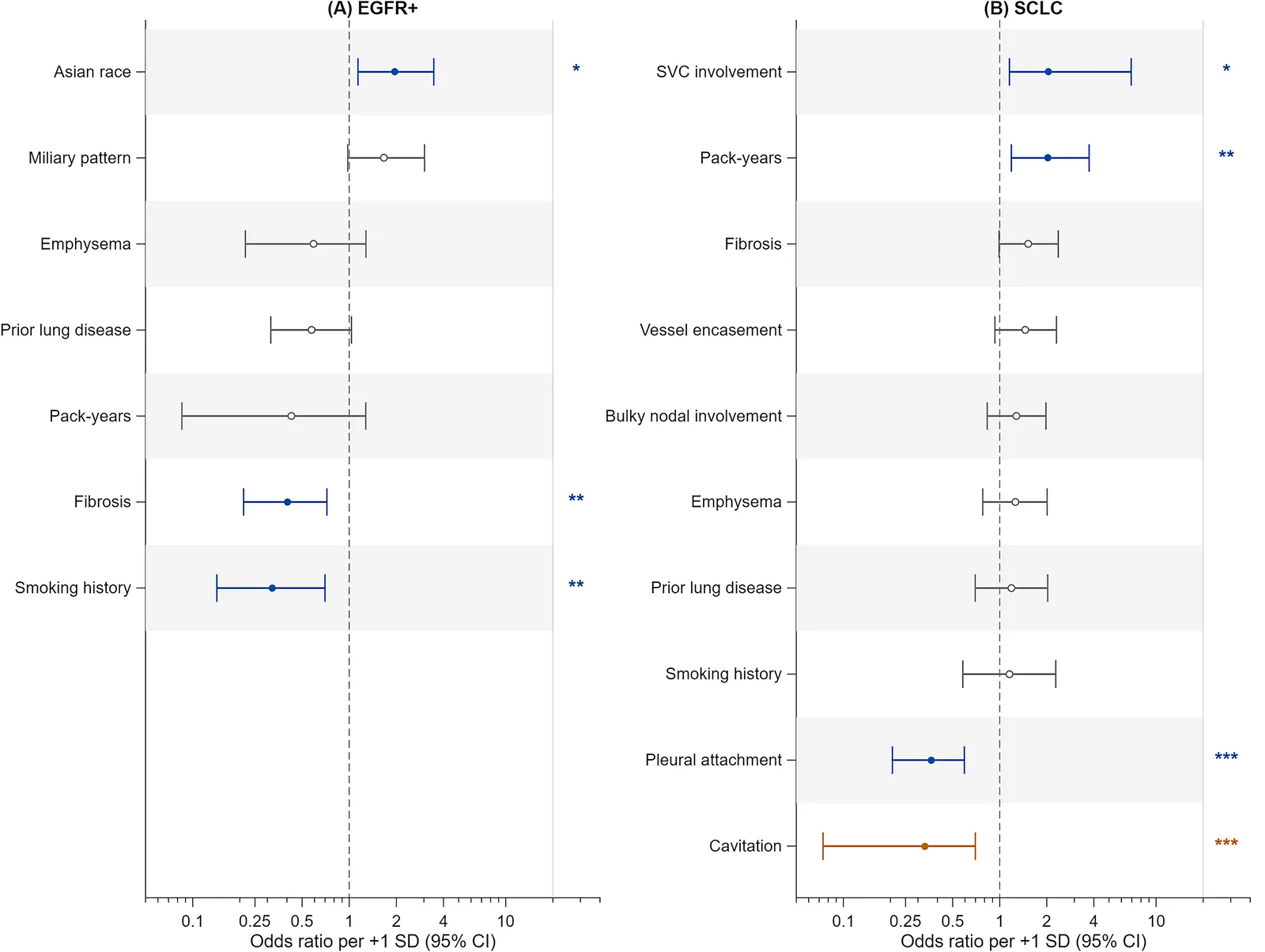

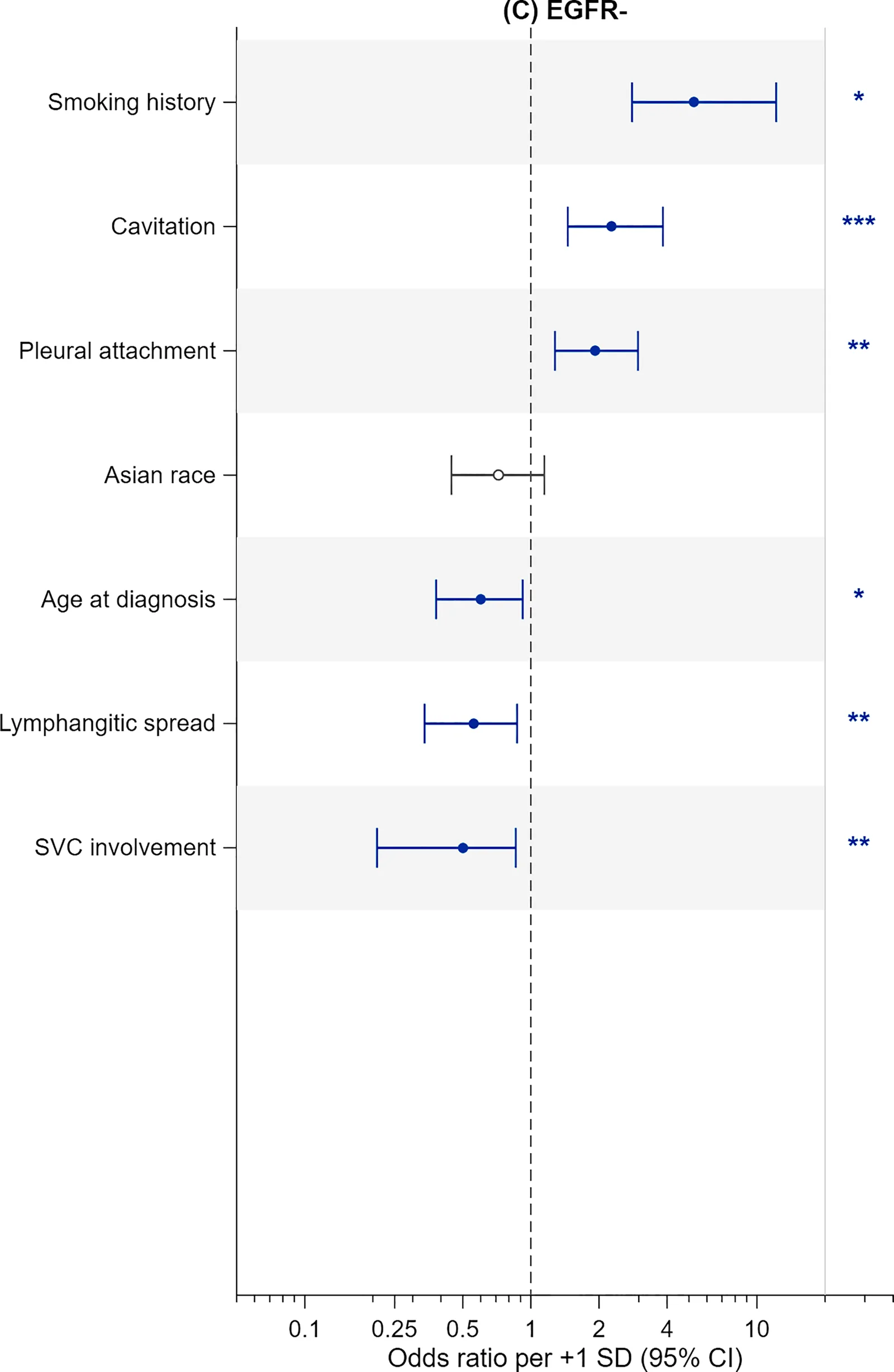
Forest plot showing odds ratios of multivariable logistic regression for clinical and imaging predictors of lung cancer subtype (**A**) EGFR+, (**B**) SCLC, and (**C**)EGFR-. Included variables are selected by LASSO penalization. Filled markers indicate statistical significance *p* < 0.05. *p < 0.05, **p < 0.01, ***p < 0.001. Orange denotes a predictor with a zero cell in this comparison, estimable only through the penalty

To determine which features contributed to subtype classification, LASSO-selected predictors were refit in multivariable logistic regression models to estimate odds ratios, demonstrated in Fig. 5. For EGFR+, Asian race and miliary pattern were associated with higher odds, while smoking history, emphysema and fibrosis were associated with lower odds. For SCLC, LASSO-selected predictors included smoking history, emphysema, SVC involvement, and pleural attachment, among others. Greater pack-year history and SVC involvement were associated with higher odds of SCLC, while pleural attachment was associated with lower odds. Finally, smoking history, cavitation, and pleural attachment were associated with higher odds of EGFR-. In contrast, older age, lymphangitic spread, and SVC involvement were associated with lower odds.

**Fig. 6:**
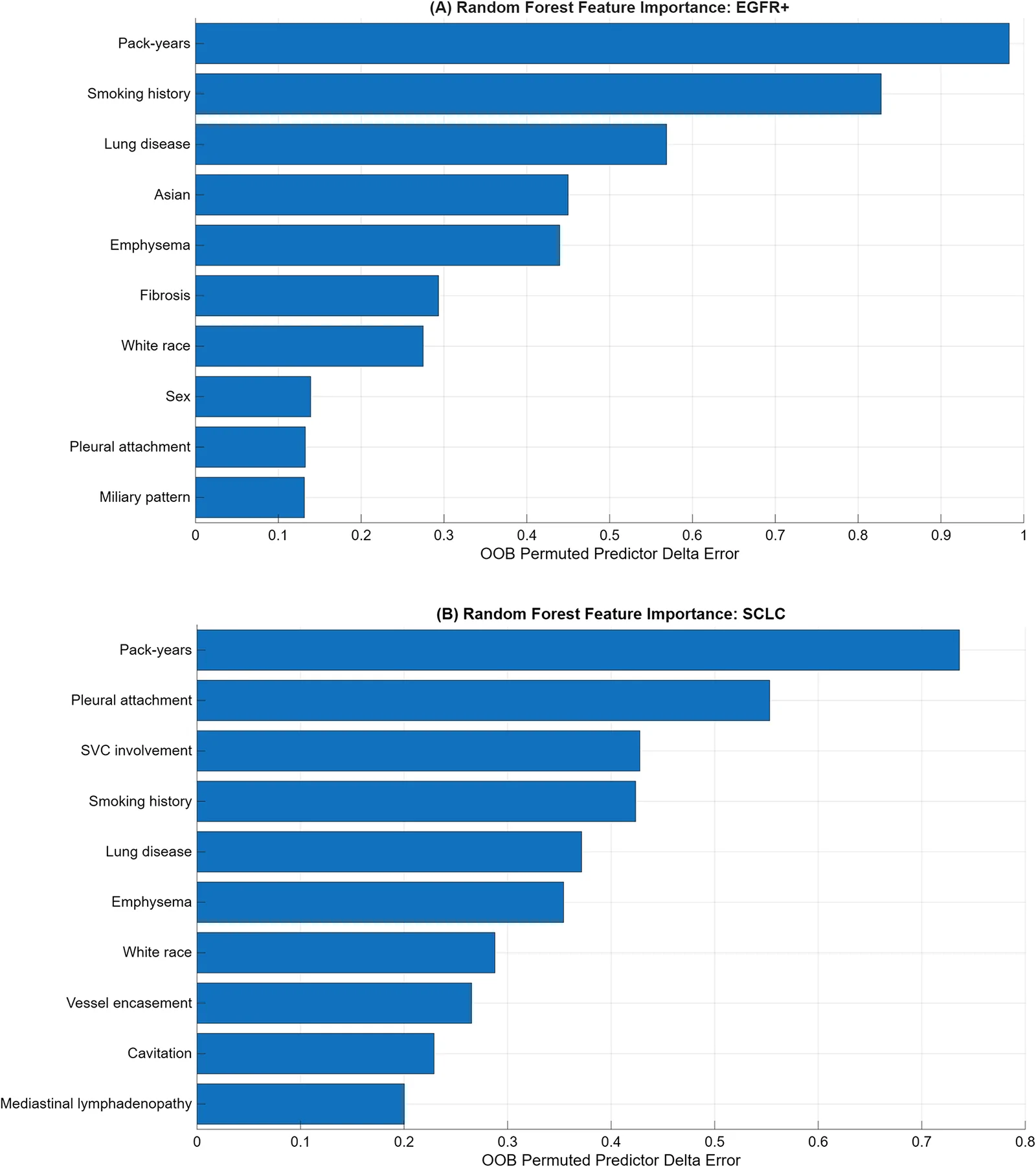

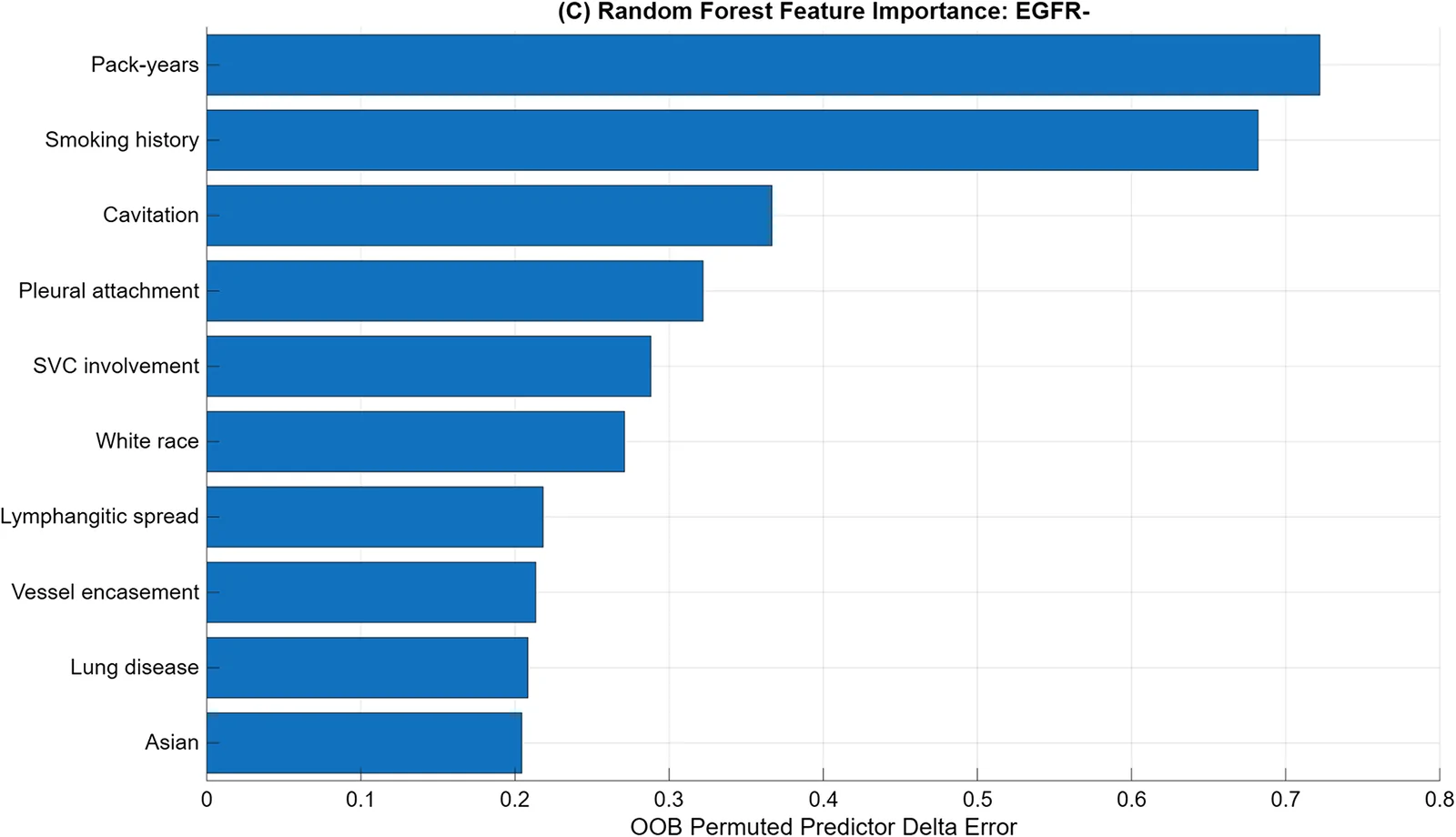
Top 10 important features of random forest model (A) EGFR+, (B) SCLC, and (C)EGFR-. Feature importance was calculated using out-of-bag permuted predictor delta error, where higher values indicate variables that contributed more strongly to model classification performance. Bars represent the relative importance of each clinical or imaging feature in the subtype-specific random forest models

In Fig. 6, random forest feature importance was evaluated using out-of-bag permuted predictor delta error, which estimates how much model error increases when each predictor is randomly shuffled and provides a measure of which variables contributed most to model performance. Importantly, it does not indicate whether a feature increased or decreased the likelihood of a given subtype. For EGFR+, the most important predictors were pack-years, smoking history, prior lung disease, Asian race, and emphysema. Additional contributors included fibrosis, white race, sex, pleural attachment, and miliary pattern. For SCLC, the most important random forest predictors were pack-years, pleural attachment, SVC involvement, smoking history, and prior lung disease. Emphysema, white race, vessel encasement, cavitation, and mediastinal lymphadenopathy also contributed to model performance to a lesser extent. For EGFR-, the highest-ranking random forest predictors were pack-years, smoking history, cavitation, pleural attachment, and SVC involvement. Other important predictors included white race, lymphangitic spread, vessel encasement, prior lung disease, and Asian race.

## Discussion

Contemporary management of brain metastases for patients with advanced lung cancer depends significantly on histological and molecular tumor features. For patients who present with synchronous brain metastatic disease, comprehensive classification introduces delays that may compromise outcomes or lead to non-optimal treatment strategies. Methods of predicting tumor subtype with immediately available information may help guide decision making in this critical period.

In this study, we developed and tested predictive models dependent only upon clinical information and reported findings from chest CT imaging, data that is readily obtainable for hospitalized patients in this clinical scenario. The goal of the models was to distinguish amongst EGFR+, SCLC, and EGFR- in patients presenting with new lung cancer diagnoses and synchronous brain metastases. Both LASSO logistic regression and random forest models were tested, and both models were trained using either clinical data alone, or clinical data supplemented with CT imaging data.

Both logistic regression and random forest models demonstrated superior performance for identifying EGFR+, with neither modeling approach demonstrating superiority. Clinical variables alone were sufficient to maximize discriminatory power for the EGFR- cohort, while the addition of CT imaging features improved discrimination of EGFR + and SCLC. The three-way prediction analysis further supports the potential clinical utility of these models. Both LASSO logistic regression and random forest models demonstrated the highest PPV for EGFR+, with PPVs exceeding 80% and NPVs approaching 90%, suggesting that patients predicted to have EGFR + may represent a particularly informative group for early multidisciplinary discussion. In contrast, SCLC classification was associated with lower PPV but relatively preserved NPV, indicating that the models may be more useful for ruling out SCLC rather than for definitively assigning small cell histology.

Decision curve analysis provided additional context for these subtype performance differences. For EGFR+, the multiclass random forest produced greater net benefit than flagging all patients or flagging no patients as EGFR+ across the full range of thresholds, indicating that a model-guided approach would outperform both default strategies. Performance for SCLC was substantially more limited and warrants more caution. Consistent with the low PPV observed for SCLC, the corresponding decision curves demonstrated added net benefit only at threshold probabilities below approximately 0.30, with net benefit becoming negative at higher thresholds. The magnitude of benefit within this window was likewise modest, corresponding to approximately five and three additional correct SCLC identifications per 100 patients evaluated at a threshold probability of 0.20 for multinomial logistic regression and multiclass random forest, respectively. The main factor that likely contributes to this limited performance is that SCLC represented only 19 of 123 patients in the testing cohort, which constrains the attainable positive predictive value at any given sensitivity and specificity, widens the confidence intervals of SCLC discrimination, and limits the precision of the net benefit estimates. Because net benefit depends on outcome prevalence, the threshold range over which benefit is observed would be expected to widen at institutions with a higher relative incidence of small cell histology. These analyses were exploratory and were not intended to establish a specific operating threshold for clinical use.

The specific feature analyses provide additional context for these performance patterns. Features such as pleural attachment and miliary pulmonary metastases were associated with EGFR+, consistent with previously described imaging phenotypes of this molecular subtype [24, 26, 27]. Similarly, features such as superior vena cava involvement and central thoracic disease distribution were associated with small cell histology [1, 28]. These findings highlight the potential value of thoracic imaging features as biomarkers of tumor histology, and taken together, suggest that readily available chest CT features provide complementary information beyond clinical variables alone and may improve early subtype estimation.

Several limitations and strengths of the study should be acknowledged. First, this single institutional retrospective study is subject to selection bias based on the geographic location, which may have a higher relative incidence of EGFR+ lung cancer compared with other regions of the United States and globe. This may inflate the positive predictive value of the model compared with regions with lower relative incidence of EGFR+ lung cancer and high incidence of smoking related lung cancer.

In addition, imaging features were derived from clinical radiology reports. While this may increase the feasibility of implementing this approach in the clinical setting, it also means that the reports were not structured in a standardized way, potentially introducing noise into the model via misidentification of the presence or absence of features. Although manual validation demonstrated high agreement between LLM derived and human annotated report features, this analysis evaluates the fidelity of information extraction from the narrative report rather than the accuracy of the underlying radiologic interpretation. Variability in radiologist terminology, reporting practices, and omission of perceived inconsequential imaging findings therefore remains a potential source of measurement error. Future work will include investigation of effects of standardization of image interpretation as well as of more quantitative but less intuitive approaches such as radiomics analysis. It is important to note, that while an LLM was used to facilitate high-throughput, consistent data extraction for the creation of a large dataset, the modeling approach described here is in no way dependent on the use of an LLM. Further, any errors introduced into the dataset by the data extraction technique are unlikely to bias the findings towards spuriously high performance metrics. Errors would rather appear as noise in the dataset, with more potential for negative than positive effects on performance.

Another limitation is the biological heterogeneity of the EGFR- group. This category includes tumors harboring other actionable oncogenic alterations, as noted by the alternative oncogenic drivers such as KRAS G12A, ERBB2, and EML4-ALK fusions identified in Supplemental Table 6. These findings raise the possibility that some of the clinical and imaging characteristics associated with EGFR- classification may reflect a phenotype of other driver mutations. The current model therefore should not be interpreted as providing a molecular diagnosis or as a substitute for definitive molecular testing, and future studies incorporating individual molecular subgroups are needed to determine whether similar approaches can distinguish among different oncogenic drivers of NSCLC.

Finally, patients were partitioned into training and testing cohorts according to the presence or absence of synchronous brain metastases. These populations may differ in tumor biology, metastatic burden, and thoracic imaging characteristics, creating the potential for covariate and broader distribution shift. Subtype-stratified analyses and covariate reweighting were performed to evaluate the impact of measured baseline differences, and model discrimination changed minimally after reweighting. However, these analyses cannot establish that the underlying populations are equivalent or exclude residual differences in unmeasured feature distributions. The size-matched analysis was also limited by small sample size and relatively few SCLC cases and should not be interpreted as an equivalence analysis. Accordingly, the present cohort partitioning and modeling framework should be considered proof-of-concept. Generalizability requires validation in an independent external cohort of patients, and the models should not currently be used as a substitute for histologic or molecular diagnosis or as a stand-alone basis for clinical treatment decisions.

## Conclusion

Predictive models integrating clinical and chest CT imaging features can discriminate among EGFR-mutated NSCLC, small cell lung cancer, and EGFR–wild-type NSCLC with encouraging accuracy in patients presenting with synchronous brain metastases, a setting in which early subtype estimation may help inform treatment decisions. In this proof-of-concept study, performance was strongest for EGFR-mutated NSCLC and more limited for SCLC, while several imaging features emerged as potentially informative biomarkers of lung cancer subtype.

These findings suggest that clinical and radiographic information available at initial presentation may provide useful adjunctive information while definitive histologic and molecular characterization is pending. The models should not replace tissue diagnosis or molecular testing, and prospective implementation studies are warranted to determine whether such predictive tools can meaningfully improve clinical decision-making and patient outcomes.

## Supplementary Information

Below is the link to the electronic supplementary material.

Supplementary Material 1

Supplementary Material 2

Supplementary Material 3

Supplementary Material 4

Supplementary Material 5

Supplementary Material 6

Supplementary Material 7

Supplementary Material 8

Supplementary Material 9

## Data Availability

No datasets were generated or analysed during the current study.

## Declarations

### Generative AI and AI-assisted technologies in the manuscript preparation process

During the preparation of this work, the author used Anthropic Claude Sonnet 4.6 for grammar proofreading. Additionally, an institutional, firewalled implementation of Anthropic Claude Sonnet 4.6 was used as part of the study methodology to extract text from deidentified radiology reports using a standardized prompt. The authors reviewed and edited the output as needed and take full responsibility for the content of the published article.
