# Supplementary material for: Predictive modeling of lung cancer subtype with integration of clinical and thoracic imaging features to guide early brain metastasis management
Source: J Neurooncol. 2026 Oct 5;180(1):5. doi: 10.1007/s11060-026-05812-z (PMC13638719; doi:10.1007/s11060-026-05812-z)
Supplement: Supplementary file 1 [file 11060_2026_5812_MOESM1_ESM.docx]

**V8 CT - Optimized Lung Cancer Feature Extraction Prompt v8**

**Primary Objective:
Extract 15 structured lung cancer imaging features from CT radiology reports with high reproducibility and accuracy for ML training data. Assemble a single-line primary output string with 15 values separated by 14 pipes for direct CSV/Excel ingestion. Generate a secondary validation log with quotes and brief rationales for all positive findings. Flag uncertain cases requiring human review.**

**Extraction Philosophy:**

- **Semantic Recognition: Capture findings using exact terms, established synonyms, clinically equivalent descriptions, or radiological phrases representing the same features.**
- **Conservative Inference: Do not infer beyond what is explicitly stated or clearly suggested. No assumptions based on external medical knowledge.**
- **Sensitivity Priority: When uncertain, err on the side of over-reporting (1) and flag for review to avoid missing potential findings. Flag indirect but potentially relevant phrasing for manual evaluation to reduce false negatives.**

**Extraction Rules:**

- **Decision Framework:**
  - **Present (1): Explicitly stated, equivalent terminology, or phrases suggesting diagnostic suspicion ("possible," "likely," "cannot exclude," "suspicious for," "questionable," "may represent," "concerning for," "cannot rule out," "suggestive of").**
  - **Absent (0): Not mentioned or explicitly ruled out.**
  - **Review Logic: If synonym is non-standard or description is uncertain/ambiguous but may suggest presence of a feature → mark as 1 in primary output and flag with "[REVIEW - reason]" in secondary output only.**
  - **NA: Information not provided or not applicable.**

**Features (1–15)**

1. **Central vs Peripheral Location (Primary Lesion Only):**
   - **0 = Not mentioned – No description of the primary lesion as central or peripheral; or when unclear indicate 0.**
   - **1 = Central – Primary lesion explicitly described as “central,” “centrally located,” “central mass”, “Hilar”, “perihilar”, mediastinal or is in a central region**
   - **2 = Peripheral – Primary lesion explicitly described as “peripheral,” “peripherally located”, “peripheral mass”, “sub pleural”, “pleural-based” or is in a peripheral region**
2. **Pleural attachment – Presence of any tumor-related pleural abnormality, excluding pleural effusion. This includes pleural attachment, thickening, nodularity, retraction, tethering, or indentation directed toward the primary tumor. Capture any descriptors such as *“pleural-based,” “subpleural,” “pleural thickening,” “pleural retraction,” “tethering,”* or *“pleural indentation/notching”*, or “abutting the pleura” that indicate direct pleural involvement by the tumor.**
3. **Cavitation / necrotic center – Central lucency or necrosis within the primary lesion.**
4. **Lymphangitic spread – Thickened interlobular septa following tumor distribution.**
5. **Endobronchial Component – Tumor extending into, invading, or growing within the lumen of a bronchus. Includes intraluminal masses or lesions causing partial or complete airway obstruction. Excludes tumors only compressing, encasing, or abutting the bronchus without clear intraluminal invasion.**
6. **Fibrosis/Scarring – Presence of fibrosis or scarring within the lungs**
7. **Miliary pattern – Numerous, uniformly distributed, tiny (1–4 mm) nodules scattered throughout both lungs, often described as “miliary nodularity,” “innumerable micronodules,” or “diffuse miliary metastases.”**
8. **Emphysema – Presence of emphysema or hyperinflation within the lung parenchyma, typically described with terms such as "bullae," "hyperinflation," or "emphysema."**
9. **T3 or T4 (Primary tumor)– Assess the largest reported tumor dimension and invaded structures. Reference AJCC 8^th^ edition staging for NSCLC and determine if tumor falls in the category T3 or T4.**
10. **Multifocal lung disease – Presence of multiple cancer-related pulmonary lesions and/or metastatic nodules within the lungs.**
11. **Mediastinal lymphadenopathy – Presence of enlarged mediastinal lymph nodes, either described as such or reported as ≥10 mm on short axis. Also includes nodes noted as suspicious for malignancy or metastasis.**
12. **Extensive/Bulky Node – Lymph nodes described as large, bulky, or massive, or those that are larger than 2 cm (20 mm) in size, regardless of their anatomical location (mediastinal, hilar, or elsewhere), should be considered bulky.**
13. **Vessel Encasement/Invasion – Tumor encasing, invading, or abutting vessels (pulmonary arteries, veins, aorta, etc.). Includes terms like "vessel encasement," "vascular invasion," or "wrapping around vessels."**
14. **Superior Vena Cava (SVC) Involvement – Compression, encasement, obstruction, or invasion of the superior vena cava by tumor. Includes terms like "SVC syndrome," "SVC compression," "SVC encasement," or "superior vena cava involvement."**
15. **Bone metastases – Presence of osseous metastases. Do NOT treat benign or incidental bone findings (e.g., bone islands, degenerative changes, benign-appearing sclerotic foci) as suspicious for bone metastases.**

**Processing Workflow:**

- **Complete Review: Read entire report including all imaging sections**
- **Direct Matching: Identify exact terms and established synonyms using definitions provided**
- **Uncertainty Handling: Apply sensitivity priority - mark uncertain findings as present (1) with review flag for secondary output**
- **Quality Control: Verify primary and secondary outputs are congruent - every value in primary output must have corresponding explanation in secondary output; Double check values before generating final output**

**Primary Output Requirements:**

- **15 values separated by 14 pipes, with no spaces.**
- **No consecutive pipes (||).**

**Secondary Output Requirements:**

- **Present features (1): Direct quote + rationale + review flag if applicable**
- **Absent features (0):**
  - **If explicitly denied: Quote denial + rationale**
  - **If not mentioned: "No mention of this feature within the report"**
- **Review cases: Quote + explanation of uncertainty + specific review category for manual verification**
- **Generate the secondary output first and then the primary output and make sure that the primary output values match the secondary**
- **Double check the primary and secondary outputs before generating to ensure that the output feature list matches the output values and that both the primary and secondary outputs are the same**

**Example Primary Output:**

**2|0|1|0|1|0|0|1|0|0|0|1|0|0|0**

**CT - REPORT**
