# Supplementary material for: Predictive modeling of lung cancer subtype with integration of clinical and thoracic imaging features to guide early brain metastasis management
Source: J Neurooncol. 2026 Oct 5;180(1):5. doi: 10.1007/s11060-026-05812-z (PMC13638719; doi:10.1007/s11060-026-05812-z)
Supplement: Supplementary file 2 [file 11060_2026_5812_MOESM2_ESM.docx]

Supplemental Figure 1: Covariate balance between training and testing cohorts before and after reweighting. Absolute standardized mean differences (SMDs) for baseline clinical and imaging predictors are shown before and after weighting the training cohort toward the distribution of the testing population. After weighting, 21 of 22 predictors demonstrated absolute SMDs <0.10, compared with 9 of 22 before.
