# Supplementary material for: Predictive modeling of lung cancer subtype with integration of clinical and thoracic imaging features to guide early brain metastasis management
Source: J Neurooncol. 2026 Oct 5;180(1):5. doi: 10.1007/s11060-026-05812-z (PMC13638719; doi:10.1007/s11060-026-05812-z)
Supplement: Supplementary file 3 [file 11060_2026_5812_MOESM3_ESM.docx]

Supplemental Table 1: Subtype-stratified distribution of baseline characteristics that differed between the overall training and testing cohorts. Prior lung disease, fibrosis, and cavitation were compared between training and testing cohorts separately within EGFR+, SCLC, and EGFR− disease using Fisher’s exact tests. No statistically significant differences were detected within individual subtype.

| Predictor | Subtype | Training | Testing | p-value |
| --- | --- | --- | --- | --- |
| Prior lung disease | EGFR+ | 9/66 (13.6%) | 8/53 (15.1%) | 1.00 |
|  | SCLC | 41/57 (71.9%) | 11/19 (57.9%) | 0.27 |
|  | EGFR- | 43/59 (72.9%) | 29/51 (56.9%) | 0.11 |
| Fibrosis | EGFR+ | 7/66 (10.6%) | 4/53 (7.5%) | 0.75 |
|  | SCLC | 21/57 (36.8%) | 5/19 (26.3%) | 0.58 |
|  | EGFR- | 17/59 (28.8%) | 9/51 (17.6%) | 0.19 |
| Cavitation | EGFR+ | 3/66 (4.5%) | 6/53 (11.3%) | 0.19 |
|  | SCLC | 0/57 (0) | 1/19 (5.3%) | 0.25 |
|  | EGFR- | 9/59 (15.3%) | 14/51 (27.5%) | 0.16 |
