# Supplementary material for: Predictive modeling of lung cancer subtype with integration of clinical and thoracic imaging features to guide early brain metastasis management
Source: J Neurooncol. 2026 Oct 5;180(1):5. doi: 10.1007/s11060-026-05812-z (PMC13638719; doi:10.1007/s11060-026-05812-z)
Supplement: Supplementary file 4 [file 11060_2026_5812_MOESM4_ESM.docx]

Supplemental table 2: Sensitivity analysis of model performance following covariate reweighting. Training observations were reweighted to approximate the baseline clinical and imaging covariate distribution of the testing cohort. Unweighted and weighted models were evaluated on the same testing cohort under matched conditions. ΔAUC represents weighted minus unweighted testing AUC.

| Model | Subtype | Unweighted AUC | Weighted AUC | Δ AUC |
| --- | --- | --- | --- | --- |
| LR | EGFR+ | 0.887 | 0.895 | +0.008 |
| LR | SCLC | 0.811 | 0.787 | −0.024 |
| LR | EGFR- | 0.801 | 0.789 | −0.012 |
| RF | EGFR+ | 0.907 | 0.911 | +0.004 |
| RF | SCLC | 0.783 | 0.780 | −0.003 |
| RF | EGFR- | 0.810 | 0.816 | +0.006 |
