# Supplementary material for: Predictive modeling of lung cancer subtype with integration of clinical and thoracic imaging features to guide early brain metastasis management
Source: J Neurooncol. 2026 Oct 5;180(1):5. doi: 10.1007/s11060-026-05812-z (PMC13638719; doi:10.1007/s11060-026-05812-z)
Supplement: Supplementary file 5 [file 11060_2026_5812_MOESM5_ESM.docx]

Supplemental table 3: Size-matched sensitivity analysis of training cohort partitioning. Testing AUCs are shown for models trained on 60 patients with brain metastases, Cohort A, versus 60 patients without brain metastases, Cohort B. P-values reflect paired DeLong comparisons between cohorts.

|  | AUC, cohort A | AUC, cohort B | p-value |
| --- | --- | --- | --- |
| EGFR+ LR | 0.850 | 0.871 | 0.464 |
| SCLC LR | 0.500 | 0.682 | 0.112 |
| EGFR- LR | 0.720 | 0.811 | 0.119 |
| EGFR+ RF | 0.869 | 0.904 | 0.173 |
| SCLC RF | 0.764 | 0.700 | 0.327 |
| EGFR- RF | 0.794 | 0.869 | 0.171 |
