# Supplementary material for: Predictive modeling of lung cancer subtype with integration of clinical and thoracic imaging features to guide early brain metastasis management
Source: J Neurooncol. 2026 Oct 5;180(1):5. doi: 10.1007/s11060-026-05812-z (PMC13638719; doi:10.1007/s11060-026-05812-z)
Supplement: Supplementary file 7 [file 11060_2026_5812_MOESM7_ESM.docx]

Supplemental Table 4: Manual validation of LLM based thoracic imaging feature extraction.

| Imaging feature | Human positive | LLM positive | Agreements | Comparisons | % Agreement | Cohen’s kappa |
| --- | --- | --- | --- | --- | --- | --- |
| **Location** | Central 13; Peripheral 13 | Central 15; Peripheral 12 | 48 | 50 | 96% | 0.937 |
| **Pleural attachment** | 23 | 21 | 48 | 50 | 96% | 0.919 |
| **Cavitation** | 3 | 3 | 50 | 50 | 100% | 1.000 |
| **Lymphangitic spread** | 15 | 13 | 48 | 50 | 96% | 0.901 |
| **Endobronchial component** | 8 | 6 | 48 | 50 | 96% | 0.834 |
| **Fibrosis** | 9 | 9 | 48 | 50 | 96% | 0.864 |
| **Miliary** | 3 | 2 | 49 | 50 | 98% | 0.790 |
| **Emphysema** | 5 | 6 | 47 | 50 | 94% | 0.694 |
| **T3 or T4** | 28 | 33 | 45 | 50 | 90% | 0.792 |
| **Multifocal** | 36 | 38 | 48 | 50 | 96% | 0.896 |
| **Mediastinal lymphadenopathy** | 25 | 23 | 48 | 50 | 96% | 0.920 |
| **Bulky nodal involvement** | 8 | 9 | 49 | 50 | 98% | 0.929 |
| **Vessel encasement** | 6 | 6 | 50 | 50 | 100% | 1.000 |
| **Superior vena cava involvement** | 1 | 1 | 50 | 50 | 100% | 1.000 |
| **Osseous Metastases** | 12 | 12 | 50 | 50 | 100% | 1.000 |
| **Overall** |  |  | **726** | **750** | **96.8%** | **0.918** |
