# Supplementary material for: Predictive modeling of lung cancer subtype with integration of clinical and thoracic imaging features to guide early brain metastasis management
Source: J Neurooncol. 2026 Oct 5;180(1):5. doi: 10.1007/s11060-026-05812-z (PMC13638719; doi:10.1007/s11060-026-05812-z)
Supplement: Supplementary file 8 [file 11060_2026_5812_MOESM8_ESM.docx]

Supplemental table 6: Alternative driver mutations identified among false positive EGFR+ predictions.

| Patient ID | Driver mutation |
| --- | --- |
| 171 | KRAS G12A |
| 181 | None |
| 200 | ERBB2 |
| 201 | None |
| 287 | EML4-ALK fusion |
| 305 | EML4-ALK fusion |
