# Supplementary material for: Predictive modeling of lung cancer subtype with integration of clinical and thoracic imaging features to guide early brain metastasis management
Source: J Neurooncol. 2026 Oct 5;180(1):5. doi: 10.1007/s11060-026-05812-z (PMC13638719; doi:10.1007/s11060-026-05812-z)
Supplement: Supplementary file 9 [file 11060_2026_5812_MOESM9_ESM.docx]

Supplemental Table 7: Machine learning model specifications and hyperparameters.

| **Parameter** | **Specification** |
| --- | --- |
| **Data preprocessing** |  |
| Missing data | Missing baseline clinical value was replaced by median imputation computed from the training cohort. Imaging predictors were complete by construction since a feature not described in the report was coded absent rather than missing. |
| Predictor scaling | All predictors were z-standardized using the mean and standard deviation of the training cohort only, with the same constants applied unchanged to the testing cohort. |
| Predictor coding | All predictors were entered as single numeric columns. Binary features were coded 0/1; tumor location was recorded on a three-level scale and entered as one numeric term. |
| **Model development and internal validation** |  |
| Data partitioning | The training and testing cohorts are disjoint. All imputation, standardization, penalty selection, and model fitting used the training cohort exclusively. The testing cohort was used solely to generate reported performance estimates. |
| Internal validation scheme | 10-fold cross-validation within the training cohort, used to select the LASSO penalty parameter. |
| Hyperparameter tuning | The LASSO penalty parameter was the only tuned quantity. All random forest settings were fixed a priori and were not tuned against cross-validated or testing cohort performance. |
| **LASSO logistic regression** |  |
| Software and function | MATLAB R2025b (The MathWorks, Natick, MA), Statistics and Machine Learning Toolbox; lassoglm. |
| Model family | Binomial distribution, logit link. Three one-versus-rest binary models per predictor set. |
| Elastic-net mixing parameter (alpha) | 1.0 (pure L1 penalty). |
| Penalty (lambda) search space | 100 values (software default) on a geometric grid with LambdaRatio = 10⁻⁶, so that the smallest penalty evaluated was 10⁻⁶ times the largest. |
| Penalty selection procedure | 10-fold cross-validation within the training cohort, with lambda chosen by the one-standard-error rule: the largest penalty whose mean cross-validated deviance lay within one standard error of the minimum. |
| Final lambda, clinical plus imaging | EGFR+ 0.052565; SCLC 0.049000; EGFR- 0.044523. For reference, the deviance-minimizing values on the same grids were 0.014971, 0.007986, and 0.019273. |
| Final lambda, clinical only | EGFR+ 0.105615; SCLC 0.130147; EGFR- 0.077804. For reference, the deviance-minimizing values on the same grids were 0.014971, 0.012138, and 0.014579. |
| Predictors retained at final lambda | Clinical plus imaging: EGFR+ 7, SCLC 10, EGFR- 7 of 22 candidates. Clinical only: EGFR+ 3, SCLC 2, EGFR- 2 of 7 candidates. Retained predictors and their refit odds ratios are shown in Figure 5. |
| Post hoc effect estimates | Predictors with nonzero LASSO coefficients were refit in an unpenalized multivariable logistic regression to obtain adjusted odds ratios per 1 SD with Wald 95% confidence intervals (Figure 5). These refit models were used for interpretation only. |
| **Random forest** |  |
| Software and function | MATLAB R2025b, Statistics and Machine Learning Toolbox; TreeBagger, Method = classification. |
| Number of trees | 500 |
| Minimum leaf size | 5 |
| Maximum tree depth | Not constrained. |
| Split criterion | Gini diminution of impurity (software default). |
| Surrogate splits | None. |
| Resampling | Bootstrap with replacement. |
| Predictors sampled per split | Square root of the number of predictors, the software default for classification: 5 of 22 for the clinical plus imaging set and 3 of 7 for the clinical only set. Split selection used PredictorSelection = allsplits. |
| Class priors and misclassification cost | Empirical class priors and a uniform cost matrix, both software defaults. |
| Variable importance | Out-of-bag permuted predictor delta error, the increase in out-of-bag prediction error after random permutation of each predictor (Figure 6). This quantifies contribution to model performance and does not indicate direction of association. |
| **Three-class classification** |  |
| Multinomial logistic regression | A single nominal multinomial model fitted on the clinical-plus-imaging predictor set, producing one joint three-class probability vector per patient. |
| Multiclass random forest | A single three-class TreeBagger model with the setting above (500 trees, minimum leaf size 5, allsplits, out-of-bag prediction and importance enabled), fitted on the clinical-plus-imaging predictor set. |
| Class assignment | Predicted probabilities across the three subtypes sum to 1 for both models, verified numerically. Each patient was assigned to the subtype with the highest predicted probability; no probability threshold was applied. |
| Three-class performance metrics | Overall accuracy, macro-averaged F1, and Cohen kappa, with per-subtype sensitivity, specificity, PPV, NPV, and F1 derived from the 3 x 3 confusion matrix (Figure 3, Supplemental Table 5). |
| **Performance evaluation** |  |
| Discrimination | AUC with bias-corrected and accelerated bootstrap 95% confidence intervals, 2000 resamples, computed with a separate fixed seed after all models had been fitted so that resampling could not perturb the random number stream used for model construction. |
| Classification metrics | Sensitivity, specificity, positive and negative predictive value, F1, overall accuracy, and Cohen kappa. |
| Model comparison | Paired DeLong tests, used to compare clinical-only against clinical-plus-imaging models and LASSO against random forest within the same testing cohort. |
| Calibration | Not formally assessed. The models are presented as discriminative tools rather than calibrated probability estimators, and no recalibration was applied. |
| Clinical utility | Exploratory decision curve analysis in the testing cohort across thresholds using the three-class model probabilities (Figure 4). |
